# Student and faculty perceptions on the rapid scale-up of medical students in Ethiopia

**DOI:** 10.1186/s12909-016-0849-0

**Published:** 2017-01-13

**Authors:** Brittney S. Mengistu, Holly Vins, Caitrin M Kelly, Daphne R. McGee, Jennifer O. Spicer, Miliard Derbew, Abebe Bekele, Damen Haile Mariam, Carlos del Rio, Henry M. Blumberg, Dawn L. Comeau

**Affiliations:** 1 Department of Behavioral Sciences and Health Education, Rollins School of Public Health, Emory University, 1518 Clifton Rd, Rm 510, Atlanta, GA 30322 USA; 2 Rollins School of Public Health, Emory University, 1518 Clifton Rd, Atlanta, GA 30322 USA; 3 Emory University School of Medicine, 1599 Clifton Rd, 6th fl, Atlanta, GA 30322 USA; 4 Emory University School of Law and Rollins School of Public Health, Emory University, 1518 Clifton Rd, Atlanta, GA 30322 USA; 5 Department of Medicine, Emory University School of Medicine, 1599 Clifton Rd, 6th fl, Atlanta, GA 30322 USA; 6 College of Health Sciences, Addis Ababa University, Black Lion Hospital Campus, Second Floor, Office No 35, PO Box 5729, Addis Ababa, Ethiopia; 7 School of Medicine, Addis Ababa University, PO Box 9086, Addis Ababa, Ethiopia; 8 School of Public Health, Addis Ababa University, PO Box 9086, Addis Ababa, Ethiopia; 9 Hubert Department of Global Health, Rollins School of Public Health and, Department of Medicine, Emory University School of Medicine, 1518 Clifton Rd #7011, Claudia Nance Rollins Bldg, Atlanta, GA 30322 USA; 10 Division of Infectious Diseases, Department of Medicine, Emory University School of Medicine and Departments of Epidemiology and Global Health Rollins School of Public Health, Emory University, 1599 Clifton Rd, 6th fl, Atlanta, GA 30322 USA

**Keywords:** Medical education, Africa, Evaluation

## Abstract

**Background:**

Ethiopia is a country of over 94 million people that has a severe physician shortage with approximately only 2.5 physicians per 100,000 persons. Recently, the Ethiopian government implemented a “flood and retain” initiative to rapidly increase the quantity of physicians in Ethiopia. Consequently, medical student enrollment at Addis Ababa University (AAU) School of Medicine increased from 100 to approximately 300–400 students per class. This study evaluated the impact of the rapid scale-up in the number of medical students on the quality of medical education at AAU and the impact of the U.S. government-funded Medical Education Partnership Initiative (MEPI) grant awarded to AAU to provide resources to strengthen the quality of medical education at AAU.

**Methods:**

Qualitative, semi-structured, in-depth interviews were conducted with 22 key informants including faculty members, administrators and medical students at AAU. The audio recordings were transcribed verbatim and interview data were analyzed with thematic analysis.

**Results:**

Four key themes emerged from the data. Overall, participants perceived a decrease in the quality of medical education at AAU due to challenges created by the rapid scale-up in the number of medical students. Positive learning environments were described as difficult to achieve due to overcrowding in classrooms and the limited numbers of textbooks. Overall, participants stated that infrastructure improvement is needed to provide adequate medical student training. The medical education initiatives implemented and funded by MEPI have provided significant resources to support the medical student curriculum but additional resources are required to accommodate a large student body.

**Conclusions:**

The unprecedented rapid scale-up of medical students has impacted multiple facets of medical education at AAU. It is important to consider the perspectives of students and faculty in order to focus future medical education policies, MEPI programming and the allocation of resources.

## Background

Ethiopia has one of the most severe physician shortages in the world with 2.5 physicians per 100,000 persons [1]. Many factors contribute to this physician shortage but the increasing rate of population growth coupled with the low retention of physicians in Ethiopia is undoubtedly important [2, 3]. The brain drain has resulted in an overwhelming number of Ethiopian physicians migrating to higher income countries in Europe, North America, or other African countries for higher salaries, improved working conditions, or better career options [4–6]. In response to the physician shortage, Ethiopia developed training programs to rapidly increase the number of non-physician healthcare workers, thus increasing coverage and lowering cost of health care [7, 8]. Though healthcare workers play a prominent role in improving health outcomes in Ethiopia [9, 10], the current physician shortage remains a barrier to meeting all healthcare needs of the population [8, 11]. In 2005, the Ethiopian government initiated a “flood and retain” initiative to rapidly increase the number of physicians in the country and counter the brain drain [12–14]. As a result, more than 20 new medical schools were created and the Ministry of Education instructed all existing public medical universities in Ethiopia to expand their enrollment [12, 15, 16]. Since the initiative’s implementation, enrollment at Addis Ababa University (AAU) School of Medicine has more than tripled, while the number of faculty, funding, and resources has not increased proportionally.

Medical education at AAU, along with all secondary education in Ethiopia, is taught in English. Under the direction of the Ministry of Education, high school students with exceptional national exam scores are distributed to undergraduate universities [16]. Students admitted into AAU School of Medicine typically complete their training within six years [16]. General science courses are attended for the first six months, prior to completing pre-clinical years 1 and 2. Pre-clinical education consists of traditional didactic lectures and laboratory sessions. Three clinical years (Year 3, Year 4, Year 5) follow the pre-clinical years. The last clinical year includes participation in a rural community health training program where students are assigned to rural areas in Ethiopia to gain clinical exposure and research skills [17–19]. Upon successful completion of qualifying examinations, students are required to complete an internship year and are referred to as “interns” (Year 6). After completing medical school, all graduates of public medical schools are required to serve in Ethiopia as a general practitioner for two to four years. This requirement was implemented by the Ministry of Health as an effort to reduce the brain drain and expand health coverage by retaining the number of physicians practicing in Ethiopia, especially in rural areas where physician shortages are most severe.

The Medical Education Partnership Initiative (MEPI), a U.S. government funded collaborative program, was created to enhance the quality of medical education and to encourage faculty retention in sub-Saharan Africa [20–22]. In 2010, AAU was one of 13 institutions awarded a 5-year MEPI grant. MEPI-Ethiopia (MEPI-E) is led by AAU and includes collaboration with three additional Ethiopian medical schools [Defense Health College, Haramaya University, and Hawassa University]. MEPI-Ethiopia (MEPI-E) has funded several initiatives to increase access to learning resources and expand training programs for students and faculty. MEPI-E has attempted to diminish textbook shortages by expanding eLearning capacity and purchasing Android touchscreen tablets preloaded with electronic medical textbooks [12, 20]. MEPI-E funding was used to purchase eGranary, a digital library with free access to websites, books, tablet computers, academic journals, and multimedia resources [12]. Additionally, MEPI-E has provided financial support for clinical students to participate in the rural community health training programs [17, 18, 23]. Lastly, MEPI-E has taken strides to improve faculty retention by providing research training, compensation for faculty overtime, and funds to compensate residents for teaching medical students [20].

Although MEPI-E has begun to address issues of the quality of medical education [20], little research has been published about the impact of the scale-up on medical students and the usefulness of MEPI initiatives from those at AAU. The lack of literature on rapid scale-up initiatives in medical education warrants qualitative inquiry to capture the voices of medical students and faculty members. In addition, the pace of the scale-up in Ethiopia has been unprecedented and provides an opportunity to assess the impact of a rapid scale-up on medical education. This qualitative study aims to explore the student, faculty and administrators’ perceptions of the rapid scale-up as a strategy to increase the number of the country’s physicians and the impact, if any, it has on the quality of medical education at AAU. Furthermore, we explored the student and faculty perceptions of the MEPI-E programs, which is critical when considering the implementation and sustainability of medical education initiatives.

## Methods

Qualitative research is commonly used to explore phenomena where little information is known [24]. By eliciting context-rich information, the emic perspective is captured, which begins the process of deductive and inductive theory development [24]. Unlike quantitative research, the sample size in qualitative research is generally small and the findings are not generalizable to the larger population [25]. However, qualitative data are saturated with meaning and experiences that would be otherwise lost in quantitative research [25] and findings are often transferable to similar study populations [26]. We conducted semi-structured, in-depth interviews with students, faculty and administrators to gather participant perceptions and experiences about the scale-up of medical students and MEPI-E initiatives. These interviews used open-ended questions to collect information about attitudes, feelings, opinions, and experiences [27–29]. The study protocol was submitted to the Emory University and AAU Institutional Review Boards. However, it was waived from the formal approval process because it was considered part of an ongoing educational evaluation and therefore did not meet the institutional definition of research.

### Participant recruitment

Participants were recruited using convenience and purposive sampling. Members of the research team at AAU assisted in establishing initial contact with faculty members, administrators, resident, and student leaders. After interviewing student leaders, additional students and interns were recruited using snowball sampling [30]. Participants were eligible for an interview if they self-identified as being affiliated with AAU as a faculty member, administrator, student, intern, or resident.

### Data collection

Four authors (B.S.M., H.V., D.R.M., and C.M.K.) conducted face-to-face interviews between June and August 2014 with the use of a semi-structured interview guide. The interview guide consisted of primarily open-ended questions about the scale-up; pre-clinical, clinical, and faculty experiences; educational resources; initiatives implemented by MEPI-E; and, general satisfaction with medical education. Interviews were arranged at a time and a location that was most convenient for the participants and lasted between 20 and 60 min. Medical education is taught in English, so it was appropriate to conduct the majority of the interviews in English; one interview required a translator. Before beginning the interviews, the interviewers read a consent script that explained the voluntary nature of the interview and the purpose of the assessment. This included explaining that the interviews were confidential and that all identifying information would be removed from the data. The participants were given the option to decline the interview or discontinue the interview should they feel uncomfortable. All of our respondents agreed to participate before the interviewers proceeded with turning on the recorder and beginning the interview. We did not collect identifying information. However, on occasion, our respondents would mention the names of colleagues, peers, programs and institutions during the interviews. These identifiers were removed from the data. All the interviews were audio-recorded, transcribed verbatim, and de-identified to ensure participant confidentiality.

### Data analysis

We used thematic analysis to examine the data, and deductive and inductive coding processes were utilized in order to identify major themes throughout the transcripts [27]. Transcripts were imported into a qualitative software program, MAXQDA 10 and 11 (VERBI Software-Consult-Sozialforschung GmbH, Berlin, Germany), which assisted in data coding, management and analysis. Two authors (B.S.M. and H.V.) independently coded each transcript to identify major themes that elicited context-rich responses, resulting in an exhaustive list of codes that emerged throughout data analysis. Deductive codes were derived from previous knowledge and topics in the interview guide, and inductive codes were developed by identifying patterns that emerged from the data [24]. Through the identification and defining of codes, we engaged in an iterative process to better understand the themes present throughout each transcript. A constant comparative approach was used to confirm the presence and meaning of codes between all responses [31]. The senior author (D. L. C.) coded a subset of transcripts to confirm the validity of codes and definitions and ensure inter-coder agreement. We reviewed the findings with Ethiopian collaborators in order to validate emerging themes in the data. Our Ethiopian collaborators provided substantial background knowledge and context to assist in understanding the perceptions surrounding the rapid scale-up of medical education.

## Results

A total of 22 participants from the AAU School of Medicine (3 administrators, 6 faculty members, 1 postgraduate medical resident, 5 pre-clinical medical students, 5 clinical medical students, and 2 “interns”) participated in this study; 10 were men and 12 were women. The amount of time affiliated with AAU ranged from one to two years for pre-clinical students and up to 17 years for faculty members. All participants were exposed to MEPI-E related activities such as serving on an advisory board, attending a sponsored activity or training, or receiving a tablet sponsored through MEPI-E (Table 1).

**Table 1:** Characteristics of study participants (*n* = 22)

Participants	Total (*n* = 22)	Male (*n* = 10)	Female (*n* = 12)	Time at AAU Medical School	Exposure to MEPI-E
Administrators	3	2	1	2 – 6 years	Works with MEPI programs
Faculty Members	6	4	2	3 – 17 years	MEPI-E Advisory Board Received MEPI-E funds Works with MEPI-E programs
Resident	1	0	1	6 years	Attended MEPI-E training
Interns	2	1	1	5 years	Participated in MEPI-E program Received tablet
Pre-Clinical Students	5	1	4	1–2 years	Received tablet
Clinical Students	5	2	3	3 –6 years	Participated in MEPI-E program Received tablet

Four thematic domains emerged during analysis that elicited context-rich responses (Table 2). All participants stated that at least some aspects of the quality of medical education had been compromised due to the large influx of medical students without proportional increases in accompanying resources and infrastructure. As a result, the majority of participants reported negative learning and teaching experiences. Participants who were aware of MEPI-E mentioned that MEPI-E had begun to address some of the effects of the scale-up at AAU

**Table 2:** Themes on the rapid scale-up of medical students at Addis Ababa University

Theme	Definition	Quote
Perceived quality of medical education	Factors that influence the ability to learn or teach medicine.	“So, when the number increased, obviously, the quality of education is influenced, because when, you see, our lives, we are too many in the lab room…the students are too many or there is a scarcity of labs microscopes.” (Student)
Need for infrastructure improvement	Describes essential resources to enhance the quality of medical education.	“We don’t have that much good facility…the library is very small, is really embarrassing to see it.” (Student)
Satisfaction with medical education	Contentment with the learning environment at AAU.	“I guess it’s very good, you know, because, you know, when I see my seniors…the senior doctors — senior students, you know, I actually respect [them] because…they know a lot and they try to help people a lot.” (Student)
Initiatives implemented by MEPI-E	Initiatives by MEPI-E that addressed the quality of medical education.	“What it [MEPI-E] has done so far is supporting the school in resources… like textbooks, the computers, and also, uh, supporting its research program.” (Intern)

### Perceived quality of medical education

The majority of participants perceived the quality of medical education to be poor because of the school’s inability to accommodate the marked increased number of medical students. Medical students expressed that the lack of lecture halls, laboratory materials, and clinical teaching hospitals have hindered their ability to adequately receive thorough training. One pre-clinical student mentioned that the number of students in the lecture hall prohibits their ability to interact with professors. Similarly, faculty members expressed challenges in facilitating class discussions or answering students’ questions during lectures due to the large class size while having limited time to cover necessary lecture materials. One faculty member stated, “Sometimes students may not get the chance to ask questions…when you go out from the [class]room, they just follow you and ask questions.”

Faculty members and students also stated that the lack of laboratory materials, such as the limited number of microscopes for histopathology and cadavers for gross anatomy, often deprives students of observing histopathology slides and participating in dissections. Faculty members often perform dissections, pathology cultures and biochemistry experiments while students are encouraged to observe rather than participate. Additionally, students stated that some of their classmates have not participated in cadaver dissections because there was not ample time for all students to participate in these procedures. Medical students expressed having to “strive for it” and find alternate methods of gaining pre-clinical laboratory exposure. One clinical student explained:

Since…our number is actually great, it’s kind of hard…to utilize the equipment we need. When we…learned in the dissecting room…one cadaver for 30 people is, that’s unimaginable, and we used to stand on chairs or tables to actually see what we were doing. And I don’t think I got the best education right then. Because I used to wait until everybody left so I can actually see or do it myself, and it was kind of hard. That, that part, it actually goes for…almost all labs…It was like, we had to…wait…like 45 min to just get to the microscope…and you kind of waste time.

Faculty members discussed strategies that AAU has implemented to increase exposure to laboratory materials, such as dividing students into small groups. One faculty member explained, “We have 360 or 400 students, so we divide them into ten groups. And the number of equipment that we have…is limited.” Participants expressed that the limited number of available resources coupled with the marked increase in the number of students has negatively impacted the quality of medical education. Faculty members were outspoken about their level of dissatisfaction with medical education. One faculty member stated:

[We went from] a class of 50 to a class of 400, we still have the same resources, the same lecture rooms…probably less number of instructors…as a result of that the quality of education is – is compromised. I don’t think we are graduating competent doctors now than (*sic*) we used to.

The quality of medical education largely influences the quality of future physicians, thus concerning many faculty members and students.

### Need for infrastructure improvement

To enhance the quality of medical education, essential resources are needed by medical students and faculty members. All participants mentioned that the current infrastructure at AAU does not accommodate the current number of medical students. The most commonly stated needed resources were teaching hospitals, libraries, larger lecture halls, and additional dormitories. Participants mentioned that increasing the number of teaching hospitals and exposure to patients would strengthen clinical training. One resident mentioned that the main teaching hospital cannot accommodate the current number of students and building additional teaching hospitals would alleviate the burden on the faculty and staff members. Additionally, one clinical student mentioned that increased exposure to clinical cases and patients would improve the quality of clinical training. For example, one faculty member explained:

If we still continue on this, uh, number of students first thing we have to build more dormitories…Second, we should think about more lecture halls, big lecture halls. You should also think about —more faculty, faculty from professors to assistants…. So we should think of more, more classrooms, more dormitories, more transport, more cars, more buses, more cafeteria, more staff, more proctors in dormitories.

Additionally, some students mentioned that the existing library is small and does not contain enough books, causing students to share hard copy books with two classmates. Another student described going to the library early to reserve a seat, so that they would have a place to study when dismissed from class. In addition to the need for new structures, several participants discussed the need to renovate existing structures such as lecture halls, laboratories, and dormitories.

### Level of satisfaction with medical education for students and faculty

Satisfaction with the learning environment at AAU varied amongst participants, as most expressed the need to address the inadequate infrastructure while others believed that some aspects of the current medical education were adequate despite the aforementioned challenges. Students reported that some dissatisfaction stemmed from the lack of resources and inability to engage in interactive learning experiences. Some clinical students mentioned that their medical education did not meet their expectations of rigor and exposure to a variety of clinical cases and patients due to the limited number of teaching hospitals. Faculty members mentioned experiencing lower job satisfaction because of the inability to interact with the majority of medical students and identify those who are struggling academically. Some faculty members mentioned a high rate of physician burnout among the clinical staff and residents due to the increased workload related to a marked increase in medical students, thus decreasing job satisfaction and career accomplishment.

Contrarily, some participants felt satisfied with the medical education they received. One student explained, “The quality is good, the professors are good. They tell us lots of information, what they know…so the quality, it’s good.” One intern expressed some degree of satisfaction with their medical education by saying, “It’s been fair to good…[but] not very nice or excellent.” Some administrators reported an increase in satisfaction with the current state of medical education because of the changes that have taken place to accommodate the number of students such as technological advancements and increased funding to the rural community health training programs. Additionally, some administrators believed that the scale-up would improve the physician shortage in Ethiopia, which is the major goal of the “flood and retain” initiative. However, all participants expressed that increasing university resources would raise the overall satisfaction of students, faculty and administrators.

### Initiatives implemented by MEPI-E

Although approximately half of participants were unfamiliar with MEPI-E by name, they all benefited from initiatives implemented by MEPI-E. All of the medical students and interns interviewed had previously received a tablet, which was funded by MEPI-E. They stated that the tablets have been helpful in increasing access to electronic textbooks and online learning resources, but the advantages have been limited. In particular, the students mentioned that the tablets have a poor battery life. One student stated, “The battery doesn’t last, so we were saying if [MEPI] can do this much, maybe they can do better and get the [better] quality [tablet]…so that we can use it forever, that would be good.”

Participants also described research activities that have been supported by MEPI-E. Clinical medical students described how the partnership between the Ethiopian Medical Students Association (EMSA) and MEPI-E has assisted in offering clinical research trainings, providing student workshops, and increasing research opportunities through MEPI-E’s collaborations. One administrator described how MEPI-E has been supporting research activities and offering financial support for students in the rural community health training programs by saying:

The [rural community health training] facility has improved with the support of MEPI. Also there were prefabricated renovations done, and a kitchen is also being constructed by AAU and kitchen facilities will be supported by MEPI. These are making the rural community health training program also favorable for students, they are being provided adequate number of beds, bed nets, and other things which are making the rural community health training program favorable for students while they stay there.

Additionally, some faculty members stated that they have received compensation from MEPI-E for teaching additional courses, which were required because of the increased numbers of students. Several faculty members also mentioned that residents were being compensated to teach student courses to alleviate the increased faculty workload. One faculty member described that involving residents in teaching medical students would allow for smaller groups of students during clinical teaching. This faculty member explained, “It would have been difficult for us alone to manage or teach the number of students without the help of residents.” A few administrators mentioned that the quality of education is improving due to the introduction of new technologies and resources funded by MEPI-E; however, MEPI-E does not have adequate funds to ensure that the rate of quality improvement is proportional to the number of students.

## Discussion

The Ethiopian Federal Ministry of Education has implemented a striking scale-up of medical students to address the severe physician shortage that exists in Ethiopia [12]. This resulted in an increased medical student class of 300–400 at AAU. Since its implementation, medical schools have been adapting to accommodate a larger enrollment of students while operating with limited resources. At AAU, MEPI-E has focused a number of its initiatives on enhancing the quality of medical education [20]. This evaluation sought to assess the perceptions of the scale-up by conducting in-depth, qualitative interviews with students, faculty and administrators. The views of these participants are important to consider when developing policies and programs aimed at improving medical education in low-income countries.

The findings of our study revealed that students and faculty perceive numerous challenges with maintaining quality education during a period of rapid enrollment of medical students. Most study respondents reported a lack of resources to accommodate the larger number of medical students at AAU. Some described suboptimal learning and teaching experiences and an absence of physical infrastructure to accommodate the increased number of students. Despite these obstacles and challenges, students expressed high levels of motivation to access educational resources by staying late in the laboratory to perform cadaver dissections, visiting the teaching faculty after class to ask questions, and reserving seats in the library. Faculty members confirm the students’ perseverance and dedication to attain an exemplary education. Faculty members and administrators also reported dedication to training a new cadre of physicians in the face of the limited resources.

In order to accommodate the rapid increase in students, MEPI-E funded several initiatives to enhance the quality of preclinical and clinical education. For example, MEPI-E purchased and distributed electronic tablets preloaded with medical textbooks as a way to decrease the student to textbook ratio and increase access to educational materials. In addition, MEPI-E funds supported new technology for classrooms, building renovations, laboratory materials, and compensation for faculty overtime. Most importantly, MEPI-E funded the improvement of Internet connectivity for students throughout the school of health sciences. Additional interactive teaching methods were employed such as online teaching, and small group didactic sessions for pre-clinical lectures and clinical bedsides. AAU introduced problem-based learning into the curriculum for the pre-clinical, first year students. The students described this curriculum as very interactive, where they work in small groups to discuss clinical vignettes and later present to the class. Participants report favorable outcomes from these initiatives but articulated the need for additional resources such as new or renovated buildings, additional textbooks, and technological advancements. However, technologies should be carefully evaluated given that several participants mentioned poor battery life resulting in limited use of their tablets.

One goal of the “flood and retain” initiative is to increase the number of Ethiopian physicians that remain in country to practice medicine and to increase the physician to population ratio. Additional resources to improve medical education, physician and faculty work conditions and incentivizing the physician workforce will strengthen this major goal of the initiative [6, 32]. New strategies for enriching faculty career development and minimizing workload should be considered. One recommendation provided by the participants in this study is to train more residents to become effective teachers and leaders [33, 34]. This approach could benefit AAU and other medical schools throughout Ethiopia.

Although this research explores the perceptions of the quality of medical education in the current environment, it does not capture in-depth faculty experiences before and after the “flood and retain” implementation. Future evaluations should consider examining pedagogical differences and faculty experiences in medical education before and after the initiative’s implementation, and their contributions, if any, to MEPI-E initiatives. In addition, quantitative exploration of student experiences is warranted. The qualitative data from this evaluation informed the development of a quantitative survey to capture student’s knowledge and attitudes regarding the “flood and retain” initiative which was implemented and analyzed after the interviews. This assessment did not explore the impact of non-physician healthcare workers in Ethiopia, but future assessments could evaluate the impact of task-shifting in tandem with the “flood and retain” initiative in improving access to quality healthcare.

Moreover, additional implications of the “flood and retain” initiative should be considered. Since completing this research, it is likely that the number of physicians has increased due to recent graduates from the scale-up at numerous medical schools across the country. Long-term, it is important to assess the quality and competency of physicians graduating from medical school. We only captured data from one medical school in Ethiopia and other Ethiopian institutions might have different experiences with resources and large student bodies. Most importantly, it is imperative to track the long-term effects of the changing educational system and the number of physicians who remain in country versus those who leave for opportunities outside of Ethiopia. Future evaluations should further explore these areas of success and investigate the potential for sustainability.

### Limitations

Our study was subject to several limitations. This assessment explored perceptions of the rapid scale-up and captured individual experiences that cannot be generalizable to a larger population. However, these findings could be transferable to other institutions experiencing a rapid increase in study body. Additionally, the use of convenience sampling to recruit faculty members and administrators, and the use of snowball sampling to recruit students, may have resulted in bias. The interviews were conducted in English and misunderstandings due to language barriers are possible. However, medical education in Ethiopia is taught in English and thus medical students and faculty are usually experienced English speakers.

## Conclusion

The rapid scale-up of medical education in Ethiopia has created a challenging working and learning environment for students and faculty. MEPI-E has provided resources to accommodate these changes. However, it is critical to continue to understand the experiences and perceptions of students, faculty and administrators so that institutions like AAU can improve medical education despite limited resources.

## Abbreviations

AAU: Addis Ababa University
EMSA: Ethiopian medical students association
MEPI: Medical education partnership initiative
MEPI-E: Medical education partnership initiative — Ethiopia
